# Systemic Chemokine Levels with “Gut-Specific” Vedolizumab in Patients with Inflammatory Bowel Disease—A Pilot Study

**DOI:** 10.3390/ijms18081827

**Published:** 2017-08-22

**Authors:** Stephanie Zwicker, Ronaldo Lira-Junior, Charlotte Höög, Sven Almer, Elisabeth A. Boström

**Affiliations:** 1 Department of Dental Medicine, Division of Periodontology, Karolinska Institutet, Alfred Nobels Allé 8, SE-141 52 Huddinge, Sweden; stephanie.zwicker@ki.se (S.Z.); ronaldo.lira.junior@ki.se (R.L.-J.); 2 Department of Medicine, Solna, Karolinska Institutet, SE-171 76 Stockholm, Sweden; charlotte.hoog@ki.se (C.H.); sven.almer@ki.se (S.A.); 3 GastroCentrum, Karolinska University Hospital, Solna, SE-171 76 Stockholm, Sweden

**Keywords:** vedolizumab, chemokines, inflammatory bowel disease, biologics

## Abstract

Vedolizumab, a gut-specific biological treatment for inflammatory bowel disease (IBD), is an antibody that binds to the α_4_β_7_ integrin and blocks T-cell migration into intestinal mucosa. We aimed to investigate chemokine levels in serum of IBD-patients treated with vedolizumab. In this pilot study, we included 11 IBD patients (8 Crohn’s disease, 3 ulcerative colitis) previously non-respondent to anti-tumor necrosis factor (TNF)-agents. Patients received vedolizumab at week 0, 2 and 6 and were evaluated for clinical efficacy at week 10. Clinical characteristics and routine laboratory parameters were obtained and patients were classified as responders or non-responders. Expression of 21 chemokines in serum was measured using Proximity Extension Assay and related to clinical outcome. At week 10, 6 out of 11 patients had clinically responded. Overall expression of CCL13 increased after treatment. In non-responders, expression of CCL13 and CXCL8 increased after treatment, and CCL20 and CXCL1 expressions were higher compared to responders. In responders, CCL28 decreased after treatment. C-reactive protein (CRP) correlated negatively with 6 chemokines before therapy, but not after therapy. Systemic CCL13 expression increases in IBD-patients after vedolizumab therapy and several chemokine levels differ between responders and non-responders. An increased CCL13-level when starting vedolizumab treatment, might indicate potential prognostic value of measuring chemokine levels when starting therapy with vedolizumab. This study provides new information on modulation of systemic chemokine levels after vedolizumab treatment.

## 1. Introduction

Inflammatory bowel disease (IBD) is a group of chronic inflammatory diseases of the intestine and primarily includes ulcerative colitis (UC), Crohn’s disease (CD), and IBD-unclassified. In 2015, it was estimated that around 2.5 million people suffer from IBD in Europe, with the highest prevalence in Scandinavia and Great Britain, and this number is predicted to further increase [1]. Characterized as a multifactorial disease, the full pathogenesis is not completely understood, but includes a genetic predisposition, environmental factors, and impaired epithelial barrier and immune response [2,3,4,5]. Changes in chemokine levels in serum have been reported in IBD [6,7], however, assessment of circulating chemokine levels following biological treatment is poorly investigated.

More than 40 members of the chemokine family are described in humans divided into the C, CC, CXC, and CXC3 subfamily. Functionally, CC chemokines are mainly responsible for the recruitment of lymphocytes, whereas CXC chemokines have the highest ability to attract neutrophils and monocytes [8,9]. Besides the recruitment of effector cells, chemokines are involved in the adhesion and migration of leukocytes across the endothelium [9,10]. To reach the site of inflammation, circulating leukocytes migrate through the endothelium in highly controlled processes, where in the interaction between integrins expressed on effector cells and adhesion molecules expressed on endothelial cells is crucial; in such interactions the effector cells migrate with a high degree of specificity. All integrins are formed by large α and small β chains. In total 14 integrin combinations are described to regulate immune cell traffic in humans [11,12,13]. The specificity of the adhesion molecules differs, whereas vascular cell adhesion molecule 1 (VCAM-1) is expressed in the gut and the brain, mucosal addressin cell adhesion molecule-1 (MAdCAM-1) is only expressed in the intestine [14].

The use of synthesized antibodies, biological agents, targeting specific components of the innate and adaptive immune system has improved the treatment of IBD. Biologics targeting tumor necrosis factor α (TNF-α), such as infliximab, adalimumab, golimumab, and certolizumab, have greatly improved IBD treatment. Anti-TNF agents aim to block cytokine signaling and to initiate inflammatory clearance through induction of apoptosis, thereby dampening the inflammatory response [15].

Up to 40% of patients treated with anti-TNF agents do not initially respond and among those who achieve remission, 30–40% lose response over time [15,16]. Therefore, new therapeutic targets have been investigated. Natalizumab, an antagonist for α_4_ integrin subunit binds both, the α_4_β_1_ and α_4_β_7_ molecules expressed on lymphocytes needed for the binding to endothelial cells via VCAM-1 and MAdCAM-1, respectively, to enter the tissue [14]. Vedolizumab (Entyvio™, Takeda, Deerfield, IL, USA), so far the only gut-specific biological treatment approved for IBD-patients, is a humanized monoclonal antibody that binds the α_4_β_7_ integrin, and thus blocks the interaction with MAdCAM-1, which is expressed on endothelial cells. In 2013, the results from GEMINI I and II, two large phase III randomized controlled trials in UC and CD, respectively, demonstrated the effectiveness of vedolizumab in inducing and maintaining remission compared to placebo [17,18]. Further, vedolizumab might restore colonic expression of genes involved in leukocyte migration in UC responders after therapy [19]. Any potential modulation of systemic cytokine and chemokine levels following vedolizumab treatment has been poorly investigated.

In this open observational pilot study, we assessed the clinical response and circulating chemokine levels following vedolizumab treatment in IBD-patients previously non-responding to anti-TNF agents.

## 2. Results

### 2.1. Clinical Findings of Inflammatory Bowel Disease-Patients after Treatment with Vedolizumab

Clinical characteristics at baseline (week 0) and follow-up (week 10) after vedolizumab treatment are presented in Table 1. At follow-up, there was an improvement in the HBI scores and decreased fecal calprotectin levels, however, these did not reach significance. Clinical response at week 10 was achieved in 6 of 11 patients (54%). When comparing the clinical parameters between responders and non-responders, there were no significant differences in age, gender, or smoking. Age at diagnosis tended to be higher in non-responders (16.8 ± 10.3 vs. 31.2 ± 14.4 years; *p* = 0.085). Baseline fecal calprotectin levels tended to be higher, whereas C-reactive protein (CRP) levels tended to be lower in responders compared to non-responders, however, this did not reach statistical significance.

### 2.2. Systemic Chemokine Levels in Inflammatory Bowel Disease-Patients before and after Treatment with Vedolizumab

**Figure 1:**
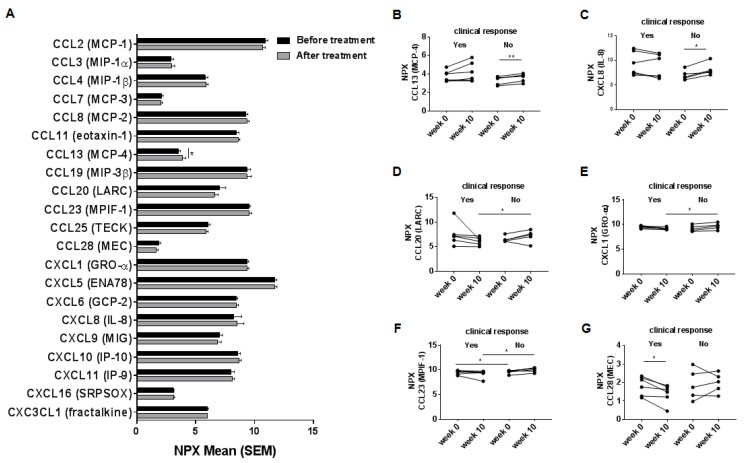
Systemic chemokine levels in 11 inflammatory bowel disease-patients previously non-responding to anti-TNF-agents before and at week 10 after induction therapy with vedolizumab. (**A**) Serum levels of in total 21 chemokines in IBD-patients at baseline (week 0) and after (week 10) treatment with vedolizumab analyzed by multiplexed proximity extension assay (PEA). Serum levels of individual chemokines; (**B**) CCL13 (MCP-4); (**C**) CXCL8 (IL-8); (**D**) CCL20 (LARC); (**E**) CXCL1 (GRO-α); (**F**) CCL23 (MPIF-1); (**G**) CCL28 (MEC) divided by the clinical response into responders and non-responders. Data represent normalized protein expression (NPX) in a log2 scale mean + SEM, * *p* < 0.05; ** *p* < 0.01, paired Student’s *t*-test; Of IBD-patients six were responders and five non-responders.

We next analyzed serum levels in a total of 21 chemokines. In the whole group of patients, an increase in CCL13 (MCP-4) expression was seen after treatment (week 10) with vedolizumab (Figure 1A). The levels of all the other analyzed chemokines remained similar. When exploring chemokine levels with respect to clinical response, only non-responders presented increased expression of CCL13 (MCP-4) and CXCL8 (IL-8) after treatment compared to baseline (Figure 1B,C). At follow-up, levels of CCL20 (LARC) and CXCL1 (GRO-α) were higher in non-responders compared to responders (Figure 1D,E), these differences were not detected at baseline. Expression of CCL23 (MPIF-1) was higher in non-responders compared to responders both at baseline and after therapy (Figure 1F). The expression of CCL28 (MEC) decreased in the responders after treatment compared to baseline (Figure 1G).

We next performed a receiver operating characteristic (ROC) analysis to test whether CCL13 expression at baseline was predictive for clinical response. The area under the curve (AUC) was 0.833 (95% CI 0.58–1.00; *p* = 0.068), indicating that the CCL13 level at baseline might be of prognostic value for response when starting therapy with vedolizumab.

### 2.3. Correlations between Systemic Chemokine Levels and Clinical and Laboratory Parameters in Inflammatory Bowel Disease-Patients before and after Treatment with Vedolizumab

**Figure 2:**
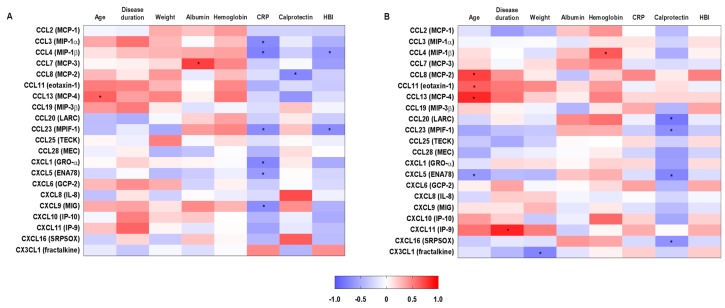
Correlations between systemic chemokine levels and clinical parameters in 11 inflammatory bowel disease-patients previously non-responding to anti-TNF-agents before and at week 10 after induction therapy with vedolizumab. Correlations between clinical and routine laboratory parameters with chemokines (**A**) at baseline (week 0) and (**B**) after treatment (week 10) with vedolizumab. * *p* < 0.05, Pearson correlation.

Correlations between clinical and laboratory parameters and chemokine levels are presented in Figure 2. At baseline, there were 11 significant correlations, with CRP levels correlating negatively with six chemokines, CCL3 (MIP-1α), CCL4 (MIP-1β), CCL23 (MPIF-1), CXCL1 (GR1-α), CXCL5 (ENA78), and CXCL9 (MIG) (Figure 2A). At follow-up, 11 significant correlations were also observed, with fecal calprotectin levels correlating negatively with the expression of CCL20 (LARC), CCL23 (MPIF-1), CXCL5 (ENA78), and CXCL16 (SRPSOX) (Figure 2B). The correlation patterns between chemokine levels and laboratory parameters, especially CRP and fecal calprotectin, were altered after treatment.

### 2.4. Chemokine Networks in Inflammatory Bowel Disease-Patients before and after Treatment with Vedolizumab

**Figure 3:**
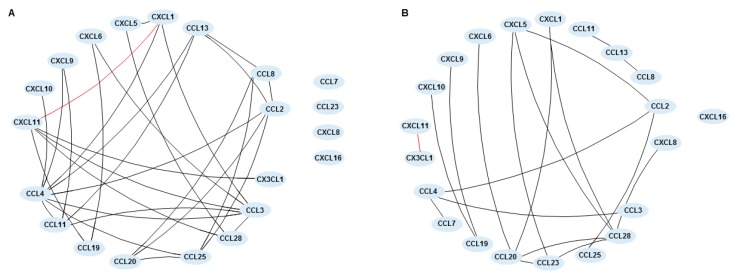
Chemokine networks in 11 inflammatory bowel disease-patients previously non-responding to anti-TNF-agents before and at week 10 after induction therapy with vedolizumab. Network was built using the correlation coefficient between each pair of chemokines, where connection lines indicate a significant correlation (*p* < 0.05) between chemokines (**A**) at baseline (week 0) and (**B**) after treatment (week 10) with vedolizumab. Each node represents a chemokine. The black line indicates a positive correlation and the red line indicates a negative correlation.

We also analyzed the chemokine network before and after treatment, which was built based on the significant correlations between chemokine pairs. At baseline, CCL4 (MIP-1β) showed the greatest number of connections with 8 significant correlations, followed by CCL3 (MIP-1α) with 6 connections. CCL11 (eotaxin-1) and CCL13 (MCP-4) (*r* = 0.843; *p* < 0.01), and CCL28 (MEC) and CXCL5 (ENA78) (*r* = 0.817; *p* < 0.01) showed the strongest correlations. The only statistically significant negative correlation was between CXCL1 (GRO-α) and CXCL11 (IP-9) (*r* = −0.620; *p* < 0.05). CCL7 (MCP-3), CCL23 (MPIF-1), CXCL8 (IL-8), and CXCL16 (SRPSOX) did not present any significant correlation with other chemokine or between themselves (Figure 3A). After treatment, CCL28 (MEC) showed the highest number of connections, with 5 significant correlations. Apart from the main cluster, 3 other groups were observed, one encompassing CCL19 (MIP-3β), CXCL9 (MIG), and CXCL10 (IP-10), CCL8 (MCP-2), CCL11 (eotaxin-1), and CCL13 (MCP-4) in another, and one with CXCL11 (IP-9) and CX3CL1 (fractalkine), which was the only negative correlation (*r* = −0.642; *p* < 0.05). The strongest correlations were seen between CCL23 (MPIF-1) and CCL28 (MEC) (*r* = 0.828; *p* < 0.01), and CCL11 (eotaxin-1) and CCL13 (MCP-4) (*r* = 0.821; *p* < 0.01). CXCL16 (SRPSOX) showed no significant correlation with other chemokines (Figure 3B).

## 3. Discussion

In this study, we investigated the clinical outcome and circulating chemokine levels following vedolizumab treatment in IBD-patients previously non-responding to anti-TNF agents. A proximity extension assay, a highly sensitive technology to quantify low protein concentrations, was applied [20,21,22] and chemokine expression level was related to clinical response to therapy. CCL13 levels increased after treatment with vedolizumab. Further, pronounced changes in the levels of several other chemokines were seen when patients were sub-grouped into responders versus non-responders, indicating that there might be a prognostic value of measuring chemokine levels when starting therapy with vedolizumab. CCL13 levels at baseline showed an AUC of 0.833 (95% CI 0.58–1.00) to predict response to induction therapy, however this result should be interpreted with caution since the sample size is small. To the best of our knowledge, this pilot study is the first study investigating systemic chemokine levels in IBD-patients before and after therapy with vedolizumab and we find support for that analyses of chemokines in serum are relevant to understand systemic changes induced by a biologic drug mainly considered to be “gut-specific”.

Vedolizumab provides clinical benefit to some patients failing anti-TNF-treatment, although the induction of clinical remission becomes evident later when compared to TNF-naïve patients [23]. From a pathophysiological point of view, CRP correlated negatively with 6 chemokines before, but not after treatment, and fecal calprotectin correlated negatively with 4 chemokines after treatment. Since median calprotectin levels were reduced, albeit not significantly, and most of the chemokine levels did not change after treatment, this could at least partially explain this correlation pattern. Sands and colleagues have shown that vedolizumab was superior to placebo in inducing clinical remission at week 10 in TNF-failure patients [24]. They found non-significant, modest improvements in CRP at week 6 and 10 after vedolizumab therapy, along with a non-significant difference in fecal calprotectin at week 6. Lower levels of fecal calprotectin were observed in UC patients treated with vedolizumab compared to placebo at week 6 [18]. Besides controlling the cell traffic, another strategy is to directly target chemokines and/or their receptors. It has been proposed that targeting the interaction between CCL25 and it receptor CCR9 might be a possible alternative to treat IBD-patients [25].

When we analyzed chemokine levels in serum before and after vedolizumab therapy, CCL13 increased after therapy. Taking into account that CCL13 induces the expression of adhesion molecules in epithelial cells and we detected an increased expression of CCL13 after therapy [26] and that CCL13 is described to recruit monocytes, eosinophils, and Th2 lymphocytes [26,27] this might explain a possible rescue mechanisms. The increased expression of adhesion molecule might allow cells from the innate immune system e.g., monocytes or neutrophils, to enter the site of inflammation in higher numbers, thus shifting the main players in the inflammatory process. This observation is of interest, since we detected significant increased levels of CCL13 in the group of non-responders as compared to the responders. However, the clinical implication of this observation needs further investigation.

When we analyzed chemokine levels according to clinical response, we found that responding patients exhibited decreased levels of CCL28 after treatment. On the opposite, in addition to elevated levels of CCL13, CXCL8 was also increased in non-responders after therapy. CCL28 is also known as mucosa-associated epithelial chemokine (MEC) [28], and its expression is associated with epithelial inflammation and recruitment of regulatory T-cells expressing CCR10 [29]. Besides its chemotactic activity, CCL28 has antimicrobial functions, and is constitutively expressed in colon [30]. CXCL8 is a potent chemoattractant for neutrophils, which in turn are an important source of CXCL8 in mucosal tissue of IBD patients [7,31]. Thus, in mucosal tissue a positive feedback loop of neutrophil attraction and CXCL8 levels occurs [32]. We therefore propose that in addition to control T-cell trafficking, the control of leukocyte influx into the intestinal mucosa might be important for achieving clinical response to vedolizumab.

A higher serum concentration of CCL20, CCL23, and CXCL1 was found in non-responders compared to responders at week 10. CCL20, described as macrophage inflammatory protein-3α (MIP-3α), binds specifically to the CCR6 receptor and has chemoattractant functions to T-cells, B-cells, and subsets of dendritic cells [31]. Systemic levels of CCL23 and CXCL1 are increased in IBD patients compared to healthy controls [7,33]. CCL23 binds to CCR1, and is chemoattractant for resting T-cells and monocytes [32,34]. CXCL1 binds to CCR2 and is chemoattractant to monocytes and neutrophils [35,36]. Taken together, we found higher serum levels of chemokines mainly known to be increased in the serum from patients with IBD, moreover, these levels were even higher in non-responders after treatment. Possibly, an increased amount of chemokines in circulation of non-responding patients might be useful to decide which patients would benefit from vedolizumab treatment beyond the induction phase. However, this need to be confirmed in larger cohorts of patients followed for longer time periods.

Comparing our results with studies investigating the chemokine expression in serum after systemic treatment with infliximab, a decreased serum level of CCL4 and CXCL8 after infliximab treatment has been reported by Sato and colleagues [37]. Further, no significant differences were observed before and after therapy in the systemic expression of CCL2, CCL3, CCL4, CXCL1, and CX3CL1 by Magnusson and colleagues in non-responders, whereas CCL4 was significantly down-regulated in responders [38].

Mouse studies showed that blocking α_4_β_7_ molecules alone did not reduce the inflammation, however, the combination of blocking l-selectin and the binding of α_4_β_7_ molecule to MAdCAM-1 was efficient in reducing inflammation in the intestine [39]. Taking our data into account one possible reason why the therapy shows side effects might be the altered systemic expression of chemokines, resulting in the recruitment of different immune cells to the site of inflammation as a rescue mechanism. In our study, non-responder patients showed higher levels of CCL13 and CXCL8, both potent chemoattractants for monocytes and neutrophils, respectively [26,27,32].

Our study has some limitations that should be taken into account when interpreting the findings. Firstly, this is a pilot study with a small sample size that could imply lack of power for some of the comparisons. However, the chemokine levels in this study could be used to calculate adequate sample sizes for future investigations. Secondly, trials are warranted either to compare the clinical and systemic/local changes of vedolizumab to a placebo control or to other drugs. Thirdly, we did not measure the drug concentration in circulation, which could be associated to the response to therapy. Despite these limitations, our study is the first one to evaluate the effects of vedolizumab therapy on the circulating levels of a large number of chemokines.

## 4. Materials and Methods

### 4.1. Study Group

11 consecutive IBD-patients with active Crohn’s disease (CD) (*n* = 8) or ulcerative colitis (UC) (*n* = 3) with an insufficient initial response, loss of response, or intolerance to anti-TNF-antibodies were included. The mean age of the study group was 31.0 ± 16.9 years; there were 3 women and 8 men, and 2 were smokers. Patients were diagnosed according to standard clinical, endoscopic, and histopathological criteria. Exclusion criteria were pregnancy and/or breastfeeding, suspicion or confirmed tuberculosis, active hepatitis B or C, any malignancy in the 5 preceding years, ongoing severe infection, or opportunistic infections. Body mass index (BMI) was calculated as the weight divided by the square of the height. Disease activity was evaluated using the Harvey-Bradshaw index (HBI) for CD and Mayo index for UC. Patients were evaluated at baseline (week 0), they received three doses (300 mg each) of vedolizumab (Entyvio^®^) intravenously at week 0, 2 and 6, and were assessed for clinical efficacy at week 10. Response or not was determined by an experienced gastroenterologist at week 10 by using all available clinical information such as patient symptoms, serum-C-reactive protein (CRP), fecal calprotectin, and endoscopic picture. Clinical symptoms were scored by using Harvey-Bradshaw index (HBI) for CD patients and Partial Mayo Score (PMS) for UC patients. Endoscopic inflammation was graded using Simple Endoscopic Score of Crohn’s disease (SES-CD) for CD and Ulcerative Colitis Endoscopic Index of Severity (UCEIS) for UC. Concomitant treatment was glucocorticosteroids in 5 patients, mesalazine (*n* = 2), azathioprine (*n* = 1) while 3 patients were taking no other drug.

Routine blood laboratory tests were obtained at baseline and at week 10, and included albumin, hemoglobin, high sensitivity CRP, and leukocyte count. Fecal calprotectin was also determined. This study was approved by the Research Ethics Committee at Linköping University (2011/201-31) and at Karolinska University Hospital Stockholm (2012/1900-31/1). The study was approved 20 July 2011 with an amendment to include Karolinska University Hospital 22 May 2013. All patients gave their informed written consent.

### 4.2. Chemokine Quantification

Chemokine expression was analyzed in serum using the Proseek^®^ Multiplex Inflammation I^96 × 96^ panel (Olink Biosience, Uppsala, Sweden). Hereby, the Proximity Extension Assay (PEA) is utilized [20,22]. Briefly, oligonucleaotide-labeled antibody pairs bind specific target proteins. Just correct matching of the antibodies allow probes to bind, which results in a polarization event and a PCR product obtained by standard real-time PCR. Relative semi-quantitative protein values are presented as normalized protein expression (NPX) in a log-2 scale, all presented data were in the range between 10^−2^ and 10^6^ pg/mL. The following chemokines were evaluated: CCL2 (MCP-1), CCL3 (MIP-1α), CCL4 (MIP-1β), CCL7 (MCP-3), CCL8 (MCP-2), CCL11 (eotaxin-1), CCL13 (MCP-4), CCL19 (MIP-3β), CCL20 (LARC), CCL23 (MPIF-1), CCL25 (TECK), CCL28 (MEC), CXCL1 (GRO-α), CXCL5 (ENA78), CXCL6 (GCP-2), CXCL8 (IL-8), CXCL9 (MIG), CXCL10 (IP-10), CXCL11 (IP-9), CXCL16 (SRPSOX), and CX3CL1 (fractalkine).

### 4.3. Statistical Analysis

Data were analyzed using Statistical Package for the Social Sciences (IBM, SPSS Inc., Chicago, IL, USA), version 20 and GraphPad Prism 6 (GraphPad Software Inc., San Diego, CA, USA). Quantitative variables are presented as mean ± standard-deviation (SD), and qualitative variables are presented as frequencies. Laboratory parameters are presented as median and interquartile range. Chemokines levels are presented as mean + standard error of the mean (SEM). The significance of differences of each parameter before and after treatment was determined by paired Student’s *t* test or Wilcoxon signed rank test. For the comparisons between responder and non-responder, unpaired Student’s *t* test or Mann-Whitney test was used according to data normality. For categorical variables, we used Fisher’s exact test. Correlations between chemokines and clinical and laboratory parameters were assessed by Pearson correlation coefficient. Chemokine networks before and after treatment were built using the significant correlation coefficients between them. Network was developed using the nwcommands (available online: http://nwcommands.org). Significance level was determined at *p* < 0.05.

## 5. Conclusions

Systemic CCL13 expression increases in IBD-patients after vedolizumab therapy and several chemokine levels differ between responders and non-responders. An increased CCL13-level when starting vedolizumab treatment might indicate potential prognostic value of measuring chemokine levels when starting therapy with vedolizumab. This study provides new information on modulation of systemic chemokine levels after vedolizumab treatment.

## Figures and Tables

**Table 1:** Clinical characteristics and laboratory parameters in 11 inflammatory bowel disease-patients previously non-responding to anti-TNF agents at baseline (week 0) and week 10 after vedolizumab therapy.

Variable	Week 0 (*n* = 11)	Week 10 (*n* = 11)	*p*-Value *
HBI	9.0 (6.0)	8.0 (8.0)	0.220
Albumin (g/L)	37.0 (2.0)	36.0 (6.0)	0.944
Hemoglobin (g/L)	130.0 (35.0)	131.0 (30.0)	0.475
Fecal calprotectin (µg/g) ^†^	2011.0 (5013.5)	191.0 (978.2)	0.069
C-reactive protein (mg/L)	3.0 (17.0)	8.0 (8.0)	0.754
Leukocyte count (×10^9^/L)	6.3 (4.9)	6.1 (2.4)	0.593

Data are represented as median (IQR). HBI: Harvey-Bradshaw index; * Wilcoxon signed rank test. ^†^ Fecal calprotectin from 3 patients was unavailable.

## Abbreviations

IBD	Inflammatory bowel disease
CD	Crohn’s disease
UC	Ulcerative colitis
HBI	Harvey-Bradshaw index
BMI	Body mass index
PGA	Physicians global assessment
CRP	C-reactive protein
TNF	Tumor necrosis factor
CCL	Chemokine (C–C motif) ligand
CXCL	Chemokine (C–X–C motif) ligand
CCR	C–C chemokine receptor
VCAM-1	Vascular cell adhesion molecule 1
MAdCAM-1	Mucosal addressin cell adhesion molecule-1
ROC	Receiver operating characteristic
AUC	Area under the curve
